# Minimally-invasive insertion strategy and in vivo evaluation of multi-shank flexible intracortical probes

**DOI:** 10.1038/s41598-021-97940-x

**Published:** 2021-09-23

**Authors:** Kagithiri Srikantharajah, Renata Medinaceli Quintela, Kerstin Doerenkamp, Björn M. Kampa, Simon Musall, Markus Rothermel, Andreas Offenhäusser

**Affiliations:** 1 Bioelectronics, Institute of Biological Information Processing-3, Forschungszentrum Jülich, Jülich, Germany; 2 Institute for Physiology and Cell Biology, University of Veterinary Medicine, Foundation, Hannover, Germany; 3 Department of Neurophysiology, Institute for Biology II, RWTH Aachen University, Aachen, Germany; 4 JARA BRAIN, Institute for Neuroscience and Medicine, Forschungszentrum Jülich, Jülich, Germany; 5 RWTH Aachen University, Aachen, Germany

**Keywords:** Experimental models of disease, Biomedical engineering

## Abstract

Chronically implanted neural probes are powerful tools to decode brain activity however, recording population and spiking activity over long periods remains a major challenge. Here, we designed and fabricated flexible intracortical Michigan-style arrays with a shank cross-section per electrode of 250 μm$^2$ utilizing the polymer paryleneC with the goal to improve the immune acceptance. As flexible neural probes are unable to penetrate the brain due to the low buckling force threshold, a tissue-friendly insertion system was developed by reducing the effective shank length. The insertion strategy enabled the implantation of the four, bare, flexible shanks up to 2 mm into the mouse brain without increasing the implantation footprint and therefore, minimizing the acute trauma. In acute recordings from the mouse somatosensory cortex and the olfactory bulb, we demonstrated that the flexible probes were able to simultaneously detect local field potentials as well as single and multi-unit activity. Additionally, the flexible arrays outperformed stiff probes with respect to yield of single unit activity. Following the successful in vivo validation, we further improved the microfabrication towards a double-metal-layer process, and were able to double the number of electrodes per shank by keeping the shank width resulting in a cross-section per electrode of 118 μm$^2$.

## Introduction

A pivotal goal in the field of neuroscience is understanding how information is processed within the brain. The objective is to decode not only the functional connections within the healthy brain, but also the dysfunctional processes that are associated with diseases. To this end, a variety of different neural probes have been developed over more than half a century. Chronically implanted neural probes are powerful tools to monitor as well as to modulate neural activity within the brain. Advancements in neurophysiological techniques and microelectromechanical systems (MEMS) technologies enabled the translation of neural interfaces from fundamental research using rodents and monkeys to clinical human applications^1,2^. Up to now, only a few neural probes were able to demonstrate significant clinical impact, namely deep brain stimulation^3^, pacemakers^4^, and cochlear implants^5^. However, these devices are mainly used for stimulation purposes as long-term monitoring of neurons (>1 year) still remains a major challenge. Different failure modes are associated with the degrading quality of neural recordings over time such as biotic mechanisms^6–8^ dictated by the interactions between the neural probe and tissue, and abiotic mechanisms^8–10^ comprising, for example, electrode related degradations and interconnection failures.

Aside from single-contact microwires^11^, there are two major types of multi-site, intracortical recording devices. Utah arrays^12^, consisting of many individual needle contacts with a single recording site at the tip, and Michigan arrays^13^ that can contain densely packed recording sites along at least one penetrating shank. While Utah arrays are currently the only intracortical devices with medical approval, multi-site and multi-shank Michigan arrays allow simultaneous recording of several neurons along the length of the neural track with a lower probe cross-section per electrode^1,2^. Traditional Michigan arrays are produced using silicon (Si), which have been shown to lose recording quality over time due to the mechanical mismatch at the device/tissue interface resulting in an enhanced foreign body reaction^1,2,14^. To improve the chronic stability, compliant probes with stress relief at the device/tissue interface have been explored within different studies including finite element modelling^15,16^ and histological studies^7,17,18^. Compliance is defined as deflection upon mechanical deformation and is associated not only with the material flexibility but also with the probe dimensions^19^. Therefore, two main approaches have been investigated to produce compliant neural devices: low Young’s modulus polymers as substrate materials^17,20–22^ and the minimization of the physical probe dimensions^23,24^. Paradoxically, intracortical probes that are compliant enough to minimize the foreign body response are unable to penetrate the tissue without mechanical reinforcement, due to their low buckling force threshold^14,19^. Therefore, several insertion strategies have been introduced to implant depth neural probes such as stiff shuttles^25,26^ and biodegradable polymer coatings^27–29^. However, such insertion systems substantially increase the spatial footprint and therefore tissue displacement during implantation. To reduce the acute trauma and improve the long-term acceptance, insertion systems that enable a precise positioning of flexible probes while preserving the small cross-section of the shanks during the implantation are still required.

In pursuit of developing flexible intracortical probes suitable for future chronic implantations, we developed Michigan-style arrays by utilizing the mechanically soft and ISO 10993 approved polymer paryleneC (PaC)^30^. To ensure high signal-to-noise ratio (SNR) recordings, the microelectrodes were coated with the conductive polymer poly(3,4-ethylenedioxythiophene) doped with poly(styrenesulfonate) (PEDOT:PSS) employing a high-throughput procedure^31^. Following probe fabrication and electrochemical characterization, we developed a tissue-friendly insertion system based on a temporary polyethylene glycol (PEG) coating. To validate the flexible probe in combination with the insertion system, we performed acute in vivo recordings in multiple mouse brain regions: the somatosensory cortex and the olfactory bulb. The device performance was evaluated using these two brain regions, responsible for sensory processing, as the physiological nature of the detected neural activities can be easily correlated to the applied stimulations^32,33^. Additionally, we took advantage of the different dorsoventral depths of the two brain regions, ranging from 0.8 to 2 mm^32,33^, to demonstrate the versatility of our flexible probes. The recording quality was characterized considering the signal amplitude as well as SNR, and compared to commercially available stiff intracortical Michigan probes. After the in vivo evaluation, we further optimized the microfabrication process and to our knowledge, were able to produce the smallest PaC based Michigan probes reported so far by employing a double-metal-layer process.

## Results

We demonstrate the microfabrication of flexible intracortical Michigan-style arrays employing PaC, and the production of microelectrodes with the low impedance coating PEDOT:PSS. Furthermore, a tissue-friendly insertion system based on a temporary PEG coating was developed, enabling the insertion of the bare, flexible shanks without increasing the implantation footprint. After successful insertion into agarose gel brain phantoms, the neural probes were implanted into mouse brains. Here, the devices were able to simultaneously detect local field potentials (LFPs), single-unit activity (SUA), and multi-unit activity (MUA). Furthermore, the flexible arrays demonstrated a similar recording quality as commercially available stiff probes or even outperformed them when considering the yield of SUA. Following the successful in vivo validation, we produced PaC based microelectrode arrays (MEAs) with a higher electrode density by establishing a double-metal-layer process to further increase the spatial resolution and to simultaneously access more cells.

### Flexible intracortical probes

**Figure 1:**
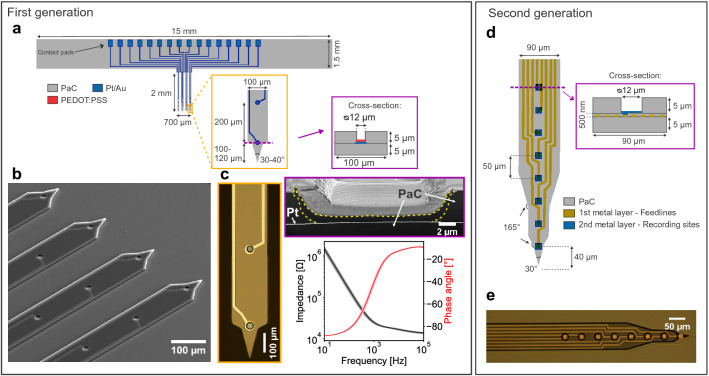
Overview of the flexible intracortical probes. First generation of devices: (**a**) Critical dimensions of the Michigan-style arrays consisting of four shanks and four microelectrodes per shank. One shank has a cross-section of 1000 μm$^2$. (**b**) Close-up SEM image of the four shanks with recording sites and metal traces consisting of a metal film sandwiched between two PaC layers. (**c**) Close-up optical image (left) of the tip of a shank and the PEDOT:PSS coating with its characteristic blue colour. FIB section of electrode coating (right top, PEDOT:PSS bordered in yellow). Bode plot (right bottom) of the spin-coated PEDOT:PSS films on a single electrode array (n = 16 electrodes, mean ± std). Second generation of devices: (**d**) A double-metal-layer process was utilized to double the number of recordings sites per shank while keeping the shank width and thickness. The layout of the bond pads is comparable to the first generation. (**e**) Optical image of one shank with eight recording sites.

The flexible intracortical probes were Michigan-style arrays, which consisted of four shanks with four microelectrodes per shank. To reduce the mechanical mismatch between the device and tissue observed for traditional Si based intracortical implants^1,2,14^, the biocompatible and low Young’s modulus polymer PaC^30^ was chosen. Additionally, a transparent, uniform and pinhole-free film can be obtained as PaC is deposited via chemical vapour deposition (CVD), which facilitates the integration into microfabrication methods. Utilizing layer-by-layer MEMS techniques, MEAs consisting of a platinum (Pt) or gold (Au) metal film sandwiched between two PaC layers were fabricated. Considering the aimed implantation strategy (see “Tissue-friendly insertion system” section), we decided to work with 5 μm thick PaC films as besides minimizing the probe dimensions, a stable handling of the probes should be guaranteed to precisely position the probe. We chose a multi-shank design to verify that our approach allows the implantation of multiple shanks simultaneously. The design was based on stiff multi-shank probes from NeuroNexus Technologies, Inc., USA (type: A4x8-5mm-100-200-177^34^) that are still state-of-the-art devices for basic research. The device design and distribution of the recording sites are shown in Fig. 1a, b. Single shanks were fabricated with a width of 100 μm and thickness of 10 μm, resulting in a cross-section of 1000 μm$^2$ and a cross-section per electrode of 250 μm$^2$ (= 1000 μm$^2$/4 electrodes). Neighbouring single shanks with a length of 2 mm and tip angle of 30–40 ° have an interdistance of 100 μm, resulting in arrays spanning 700 μm. The shanks exhibit an active length of 700 μm as the recording sites with a geometrical surface area (GSA) of 113 μm$^2$ were aligned along the shanks with a pitch of 200 μm.

To ensure high SNR recordings, a low impedance coating was established employing the soft conductive polymer PEDOT:PSS^31,35^. This conductive polymer crosslinked with ethylene glycol (EG), 3-glycidoxypropyltrimethoxysilane (GOPS), and dodecylbenzene-sulfonic acid (DBSA) was spin-coated onto the recording sites utilizing a PaC sacrificial layer. This procedure needs additional fabrication steps to establish the electrode coating however, it is a high throughput method resulting in ready-to use probes in contrast to electrochemical deposition of PEDOT:PSS, where probes are coated individually. The resulting blue coating with a thickness of 700–750 nm had a mean impedance of 2.93 ± 0.73 M$\Omega \cdot$μm$^2$ at 1 kHz (n = 125, mean ± standard deviation (std)) (see Fig. 1c). The PEDOT:PSS films caused a significant reduction in impedance compared to bare Pt or Au microelectrodes, which were characterized before covering them with PEDOT:PSS (see Supplementary Fig. S1). A representative bode plot of the PEDOT:PSS covered microelectrodes is shown in Fig. 1c.

### Tissue-friendly insertion system

**Figure 2:**
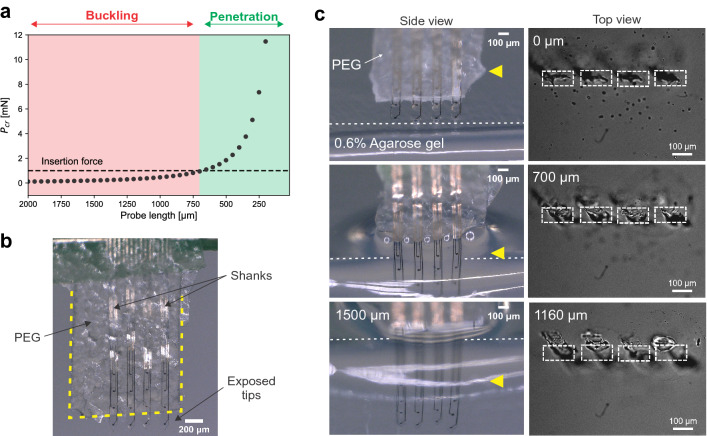
Tissue-friendly insertion system by reducing the effective length. (**a**) Buckling force threshold P$_{cr}$ as a function of probe length for the PaC based shanks with width of 100 μm, thickness of 10  μm and a Young’s modulus of 2.76 GPa^30^. The minimum insertion force of 1 mN needs to be withstand by the probe for penetration of the tissue. (**b**) Array with a PEG coating (boarded in yellow) leaving the first 200 μm from the tip exposed to temporally reduce the effective shank length. (**c**) Series of optical images showing the successful insertion of the flexible probes (see side view) using the tissue-friendly insertion system, which minimizes the implantation footprints to the dimensions of the shanks (see top view). Dashed line indicates surface of agarose gel. As the neural probe was inserted at an angle, above a penetration depth of 700 μm a slight shift was observed in the insertion holes. Side and top view were obtained from two different insertions.

Due to the low buckling force threshold, flexible probes are not able to penetrate the tissue without mechanical reinforcement (see Supplementary Fig. S2). To introduce soft and flexible shanks into the brain while minimizing the implantation footprint, a tissue-friendly insertion system was developed considering the critical load for buckling $P_{cr}$^36,37^. Single intracortical shanks of the Michigan-style arrays were assumed as fixed-pinned columns as the shanks are fixed from the top by the connector and the micromanipulator, and pinned from the tip as soon as they contact the tissue. The Euler’s formula is commonly used to compute the critical buckling load however, it only applies for long columns. Therefore, the slenderness ratio $\lambda$ (see Eq. 1) of the flexible shanks was first determined. With a slenderness ratio $\lambda$ of 490, the single shanks of the flexible array were classified as long columns^36,37^. According to the Euler’s formula (see Eq. 2), these long columns with a width of 100 μm, a thickness of 10 μm, a length of 2 mm, and a Young’s modulus of 2.76 GPa^30^ have a buckling force threshold of 0.16 mN (Fig. 2a). As rigid probes experienced forces between 0.5–1.0 mN during insertion after dura removal^38,39^, it was assumed that flexible shanks should withstand a minimum insertion force of 1 mN for a successful insertion.

From the Euler’s formula (see Eq. 2), it can be deduced that shank length plays a key role when considering buckling as the buckling force threshold is proportional to 1/L$^2$. To introduce the flexible shanks into the brain, the PaC based arrays were coated with PEG to reduce the effective length of the shanks resulting in an increased buckling force threshold. The buckling force threshold $P_{cr}$ of the shanks exceeds the minimum insertion force of 1 mN when the length is shortened to at least 700 μm (Fig. 2a). To ensure a successful insertion of the bare shanks, we decided to use an effective length of 200–300 μm, which has a $P_{cr}$ of 5–11 mN (Fig. 2a). From theory to practice, shanks with a length of 300 μm were produced and successfully inserted into gel brain phantoms (see Supplementary Fig. S2). Based on these results, the 2 mm long shanks were covered with 120 μm thick PEG (including the thickness of the shanks), leaving the first 200–300 μm from the tip exposed (Fig. 2b).

Before in vivo implantations, the insertion of the neural probes coated with PEG was tested using 0.6% (w/v in 0.1 M PBS) agarose gel brain phantoms. This concentration is commonly used as the gels demonstrate mechanical properties comparable to mammalians’ brains^40^. The array was moved with a speed of 7.5 μm/s towards the gel until the exposed shank tips were inserted and the PEG coating reached the gel surface. The PEG dissolved when in contact with saline that covered the gel. While the PEG gradually dissolved, the array was further inserted with 100–200 μm steps until at least all recording sites were placed in the gel. Flexible intracortical probes with a shank cross-section of 1000 μm$^2$ could be inserted up to 2 mm into the agarose gel (Fig. 2c (Side view)). In contrast to traditional approaches^27–29^ (see Supplementary Table S1), where the neural probe is inserted into the tissue with the entire shuttle system, our approach includes only the cross-section of the single shanks without any additional damage originating from the insertion system (Fig. 2 c (Top view)).

### Acute in vivo validation

The flexible intracortical probes and the insertion system were then validated in acute in vivo experiments. To confirm that the devices can be used in various brain regions, we performed neural recordings from the mouse barrel cortex with a dorsoventral depth of 0.8 mm as well as from the olfactory bulb with a dorsoventral depth of 2 mm (see Supplementary Fig. S3). We first conducted a craniotomy to expose the desired recording regions. Regardless of the chosen recording location, the craniotomy had a diameter of roughly 3 mm. Flexible neural probes are not able to penetrate the dura mater with a Young’s modulus of 0.4–1.2 MPa^14^, even when mechanically reinforced during insertion. Therefore, a durotomy was additionally performed to facilitate probe insertion. For olfactory bulb recordings, the dura was completely removed to gain access to the whole dorsal surface, maximizing the probability of finding odor-evoked mitral cell activity. In the barrel cortex, slits were made in the dura that served as entry points for the shanks. Exemplary preparations are shown in Supplementary Fig. S3. The implantations were comparable to the insertions into the agarose gel brain phantoms (see above).

**Figure 3:**
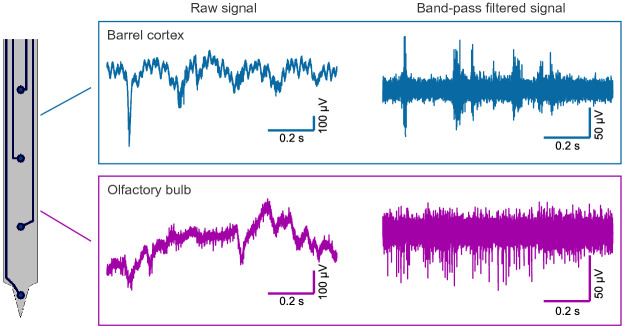
Representative electrophysiological recordings using the flexible neural probes. 1 s raw and band-pass filtered traces (5th order Butterworth band-pass, 300 Hz–10 kHz) from the mouse barrel cortex and the olfactory bulb.

**Figure 4:**
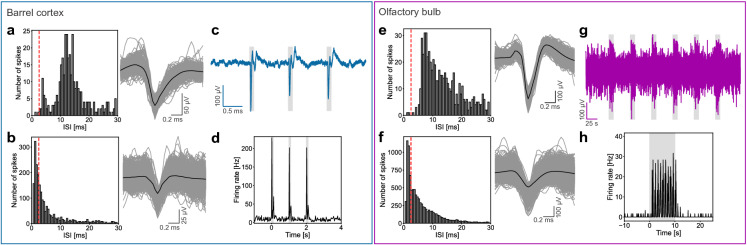
Representative examples for detected signals as well as for high correlation between stimulus presentation and neural activity recorded with the flexible intracortical probes from the barrel cortex and the olfactory bulb. The interspike interval (ISI) distributions and superimposed spike waveforms of isolated spikes for single units (**a**, **e**) and multi-units (**b**, **f**). Units where less than 1% of the ISIs were below 2.5 ms (red dashed line) were classified as single units. The mean waveforms are represented by the black lines. The LFPs (**c**, **g**) and the peri-stimulus time histograms (PSTHs) of unit activity (**d**, **h**) in response to tactile (barrel cortex) or odorant (olfactory bulb) stimulations. For the recordings from the barrel cortex (**c**), the responses are shown by averaging the raw signal over the 50 stimulation trials where as for the olfactory bulb, the raw signal is depicted for one entire stimulation trial (**g**). The stimulation pulses are represented by grey boxes.

The successful implantation of the flexible arrays were confirmed by postmortem reconstruction of the penetration tracks using DiI (see Supplementary Fig. S4). Multichannel recordings were achieved from both brain regions (see Fig. 3). Recordings from three flexible arrays inserted into the barrel cortex and six into the olfactory bulb were analysed. The recording performance of the flexible probes was determined utilizing the quality metrics signal amplitude and SNR (see Eq. 3). The spike extraction resulted in 204 and 270 units obtained from the barrel cortex and the olfactory bulb, respectively. After extracting the spikes, the clusters were distinguished into single units (SUs) and multi-units (MUs). Units where less than 1 % of the interspike intervals (ISIs) were below the refractory period of 2.5 ms were classified as SUs. In Fig. 4, the ISIs and the superimposed waveforms of representative SUs and MUs are shown for both brain regions. The recorded spiking and LFP signals were highly correlated to tactile and odorant stimulation, thus confirming that they accurately reflected the neural activity of sensory neurons (see Fig. 4).

Moreover, the performance of the flexible devices was compared to commercially available stiff Michigan probes with a single shank (ATLAS Neuroengineering bvba, Belgium or NeuroNexus Technologies, Inc., USA). In the Supplementary Table S2, an overview of the stiff devices’ critical dimensions is provided. A summary of the parameters extracted from the recordings using both neural probes grouped according to the two different applications is shown in Table 1. For the barrel cortex, a comparable performance was observed considering yield of SUA (*p* = 0.949, chi-squared test) and SNRs of the SUA (*p* = 0.788, Mann-Whitney-U-Test). When implanted into the olfactory bulb, significant differences in the performance were observed for flexible arrays compared to stiff devices. A significantly higher SU yield was achieved with the flexible arrays (*p* $\le$ 0.0001, chi-squared test). However, higher amplitudes and SNRs were recorded with the stiff devices (*p* $\le$ 0.0001, Mann-Whitney-U-Test). Overall, the flexible intracortical devices with a shank cross-section per electrode of 250 μm$^2$ could be successfully inserted into target brain regions such as the barrel cortex and the olfactory bulb as proven by recordings of spontaneous and evoked activity as well as histological reconstruction of the penetrations. For both applications, the flexible neural probes comprising recording sites with a GSA of 113 μm$^2$ were able to simultaneously record LFPs, SUA, and MUA. Furthermore, the recording performance of the flexible probes was comparable to the stiff devices (see Table 1, barrel cortex) or even superior considering the yield of SUA (see Table 1, olfactory bulb).

### Improvement towards high-density probes

One main advantage of flexible intracortical probes compared to stiff devices, besides the lower Young’s modulus, is that smaller footprints can be achieved. Our flexible probes with a shank cross-section of 1000 μm$^2$ are much smaller compared to the commercially available stiff devices as well as most other previously reported PaC based Michigan arrays (see Supplementary Table S3). To compare the density of recording sites between different intracortical probes, the ratio between shank cross-section and number of electrodes per shank is commonly used. When considering the cross-section per electrode, similar or lower densities were reached for the stiff devices compared to our flexible probes.

High-density MEAs have been developed within the last years to simultaneously access more cells and to maximize spatial resolution. After the in vivo validation of the flexible arrays, we further optimized the microfabrication to increase the electrode density per shank by taking into account that the metal traces mainly influence the shank width. To this end, we employed optical lithography in combination with a double-metal-layer process (or via design^23^) and were able to double the number of electrodes per shank without increasing the shank cross-section (see Fig. 1d, e). The double-metal-layer process was started with the deposition of a 5 μm thin PaC film as substrate layer. In contrast to the first generation, the fabrication process was further optimized enabling the patterning of 5 μm wide feedlines with an interdistance of 5 μm. After evaporating the metal film and structuring the metal traces, a 500 nm thin PaC film was deposited and openings were dry etched into this layer over the feedline ends. In the second metallization step, the recording sites and the bond pads were defined. After depositing a third PaC film as passivation layer, the probe shapes were patterned and the recording sites were exposed within the final etching step. This second generation of probes resulted in a shank cross-section of 945 μm$^2$ (= 90$\cdot$10.5 μm$^2$) and a cross-section per electrode of 118 μm$^2$ (= 945 μm$^2$/8 electrodes). Single PaC based shanks would therefore displace less than half of the tissue compared to the Si based shanks, which might further facilitate a seamless biointegration. To our knowledge, these are the smallest probes with a 38–76 % lower cross-section than previously developed flexible devices when considering only PaC based Michigan-style probes. Additionally, our flexible probes exhibited one of the smallest cross-section per electrode reported so far (see Supplementary Table S3). One probe consisting of 32 Au microelectrodes with a GSA of 144 μm$^2$ had a mean impedance of 381.24 ± 80.65 M$\Omega \cdot$μm$^2$ at 1 kHz.

## Discussion

A successful MEMS fabrication allowing the batch processing of PaC based multi-shank, multi-site probes was established. During the microfabrication process, the electron beam evaporation of 100 nm Pt on PaC films lead to a micro-cracked morphology (see Supplementary Fig. S5). In contrast, the evaporation of 100 nm Au on PaC did not induce any cracking. High temperature processes like evaporation of high melting point metals such as Pt can lead to thermal stress and therefore, cracking due to the mismatch in the thermal coefficients of expansion between metals and polymers^41^. In contrast to sputtered Pt^22,41^, no stress induced bending was observed for the flexible shanks after releasing them from the carrier wafer, which could hinder subsequent insertion. Additionally, the electrochemical measurements revealed that the impedances were comparable for Pt and Au microelectrodes (see Supplementary Fig. S1). To avoid cracking of the Pt film, a stepwise deposition with cooling steps in-between might be a good alternative^42^.

**Table 1:** Summary of single-unit activity (SUA) obtained from flexible and stiff intracortical probes grouped according to the two applications.

Parameters	Barrel cortex			Olfactory bulb		
	Flexible probe	Stiff probe	*p*-value	Flexible probe	Stiff probe	*p*-value
Total number of separated units	204	195		270	282	
Yield of SUA [%]	27 (55/204)	27 (53/195)	n.s.d.	34 (92/270)	13 (37/282)	****
Peak-to-peak amplitude [μV]	47.6 ± 4.6 (n = 55)	40.9 ± 2.6 (n = 53)	n.s.d.	99.6 ± 8.0 (n = 92)	132.8 ± 26.0 (n = 37)	****
RMS noise [μV]	7.4 ± 0.4 (n = 55)	7.1 ± 0.3 (n = 53)	n.s.d.	15.2 ± 1.0 (n = 92)	8.8 ± 1.1 (n = 37)	***
SNR	2.9 ± 0.3 (n = 55)	2.7 ± 0.2 (n = 53)	n.s.d.	2.9 ± 0.1 (n = 92)	6.3 ± 2.9 (n = 37)	****

Amplitudes, root-mean-square (RMS) noises and signal-to-noise ratios (SNRs), depicted as median ± 95 %CI, were compared employing the Mann-Whitney-U Test whereas the yield of SUA were compared using the chi-square test (*** for *p* ≤ 0.001, **** for *p* ≤ 0.0001). n.s.d = no significant difference.

To ensure high spatial resolution, the area of the recording sites was restricted to 113 μm$^2$. Such small recording sites exhibit high impedances and consequently high thermal noises, which impede high SNR recordings^43^. To overcome this issue, a high-throughput procedure was established to coat the recording sites with spin-coated PEDOT:PSS. As PEDOT:PSS exhibits both electronic and ionic conductivity involving the entire polymer volume in charge transport processes, it has a considerably lower impedance than bare metal microelectrodes^44,45^. With the low impedance coating, the median root-mean-square (RMS) noise across microelectrodes with a GSA of 113 μm$^2$ was 7.4 μV and 15.2 μV after implantation into the barrel cortex and olfactory bulb, respectively (see Table 1). The noises were similar or higher compared to the iridium oxide electrodes of the stiff devices however, these electrodes were four times bigger than the electrodes of the flexible probes (see Supplementary Table S2).

Several approaches have been proposed to temporarily or partially stiffen the flexible devices during implantation such as stiff shuttles^25,26^ and biodegradable polymer coatings^27–29^. However, such traditional strategies have the major drawback that they significantly increase the implantation footprint (see Supplementary Table S1). To reduce insertion trauma and tissue damage, the implantation footprint needs to be minimized. To this end, a tissue-friendly insertion system was developed, which was successfully validated in implantation experiments into agarose gel brain phantoms as well as mouse brains. The synthetic polymer PEG was used to mechanically reinforce the flexible shanks. As PEG is available over a wide range of molecular weights^46^ and the coating thickness can be varied without increasing the implantation footprint, the dissolution time can be tuned to fit the surgical procedure and the desired implantation time. Additionally, the coating procedure is easily scalable to multiple shanks. The length of the exposed tips can be matched to the shank dimensions to obtain a certain buckling force threshold. Furthermore, our PEG-based approach allows the production of ready-to-use devices where the coating is established on a wafer-scale before releasing the probes. One way to accomplish such a high-throughput process can be the production of a lithographically patterned PDMS mold^47^ on top of the flexible probes.

The flexible arrays could be successfully inserted into the mouse barrel cortex as well as the olfactory bulb. However, as the mouse cortex is permeated with blood vessels, probe insertion into the cortex can be complicated by bleeding. This requires rinsing the tissue with saline, which can in turn result in a faster dissolution of PEG. To increase the robustness of the insertion system, it might be beneficial to not only apply the biodegradable coating on top of the shanks rather to enclose the shanks with the coating^22^. In contrast to the cortex, the insertion into the olfactory bulb was straight forward. However, tissue dimpling, defined as temporary brain compression prior to the penetration^48^, was more pronounced when introducing the multi-shank flexible probes into the olfactory bulb. This might be related to the fact that the dura stabilizing the tissue was removed.

The quality of the recorded traces was sufficient to record spontaneous and evoked activity with high spatiotemporal resolution. When comparing both neural devices, significantly more SUs were recorded from the olfactory bulb using the flexible arrays. To maximize spatial resolution, the recording sites should have a similar size to the target cells^43^. Our flexible arrays with a GSA of 113 μm$^2$ were specifically designed to enable the recording of SUA at a high spatial resolution and were four times smaller compared to the electrodes of the stiff probes (see Supplementary Table S2). Despite the smaller GSA of the flexible probes, a simultaneous detection of LFPs, SUA, and MUA from the barrel cortex and the olfactory bulb was feasible using the flexible neural probes. Furthermore, for the olfactory bulb, significantly higher signal amplitudes and SNRs were detected with the stiff Michigan probes (see Table 1). This difference might be a consequence of probe design (single shank versus four shanks). The signal amplitude strongly depends on reaching the target neurons. The mitral/tufted cells, the principal cells in the olfactory bulb, were targeted laterally when using the stiff devices (see Supplementary Fig. S3). However, due to space restrictions, it was not possible to position the multi-shank flexible arrays spanning 700 μm as far lateral as the stiff probes. This probably resulted in a higher distance between the recording sites and the target cells, and therefore lower signal amplitudes and SNRs^43^. In contrast to the olfactory bulb, it was feasible to insert the multi-shank flexible probes as well as the single shank stiff devices into nearby areas in the barrel cortex. Therefore, similar cell populations could be targeted with both probes resulting in comparable parameters (see Table 1). Following this proof-of-concept evaluation, these differences in performance emphasize the need to design neural probes that are customized to the application including target brain region and surgical procedure to obtain the best recordings possible. For example, a single shank probe with a lower electrode pitch might be beneficial to reach the mitral cell layer of the olfactory bulb more precisely.

With the aim to further increase the channel count per shank, we established a double-metal-layer process and were able to half the cross-section per electrode from 250 μm$^2$ to 118 μm$^2$. However, the high-throughput procedure for covering the microelectrodes with PEDOT:PSS was not achieved for the second generation of devices. Combining the double-metal-layer process with the wafer-scale spin-coating process requires in total four PaC layers (substrate, passivation, inter-, and sacrificial layer). As the entire wafer is covered with PaC during the CVD, small defects in the layers introduced in the subsequent fabrication steps can already cause a complete delamination. This sensitivity to failure increases with increasing number of PaC films. Furthermore, a major challenge when down scaling neural probes is the increase in electrical crosstalk between nearby interconnections and recording sites due to reduced distance. Therefore, further optimization of the fabrication process and an in-depth characterization is needed before using the second generation of devices for in vivo recordings.

In summary, the first generation of flexible intracortical arrays were successfully inserted into the mouse barrel cortex and the olfactory bulb. Furthermore, the recording quality of the flexible probes was at least as well as the performance of commercially available stiff devices. Based on these results, we believe that the proposed flexible intracortical probes will be suitable for future chronic implantations, with the potential to perform more stably in contrast to stiff devices due to reduced foreign body responses as shown in previous works^7,17,18^. However, additional steps like characterization of the PaC films and the PEDOT:PSS coatings in regard to long-term stability are needed before going for routine chronic implantations.

## Conclusion

Flexible intracortical Michigan arrays were designed, fabricated, electrochemically characterized, and validated in acute in vivo experiments. Compliance was achieved by producing neural probes based on the mechanically soft polymer PaC and by reducing probe dimensions resulting in single shanks with a cross-section per electrode of 250 μm$^2$. To obtain high quality recordings, the microelectrodes were covered with spin-coated PEDOT:PSS, which had a significantly lower impedance than bare metal microelectrodes. To implant the neural probes, a PEG coating was used to increase the buckling force threshold above the minimum insertion force by temporarily reducing the effective shank length. Mechanically reinforced by the developed tissue-friendly insertion system, the flexible arrays were successfully inserted into brain phantoms as well as mouse brains up to a dorsoventral depth of 2 mm. Multichannel recordings of spontaneous and evoked activities were obtained from the mouse somatosensory cortex as well as the olfactory bulb, yielding robust neural signals with a median SNR of 2.9 for both brain regions. The flexible probes comprising recording sites with a GSA of 113 μm$^2$ were able to simultaneously detect LFPs, SUA, and MUA. Additionally, the flexible arrays outperformed the commercially available stiff devices with regard to the yield of SUA. Based on these promising results, the flexible intracortical arrays were further optimized with regard to electrode density and to our knowledge, the smallest PaC based Michigan-style probes were produced with a cross-section of 945 μm$^2$ resulting in a cross-section per electrode of 118 μm$^2$. In the next steps, the flexible probes will be further optimized for chronic implantations and explored as a possible solution to overcome the challenges associated with the long-term monitoring of neural activity.

## Methods

### Microfabrication of paryleneC probes

The MEAs were produced employing PaC as substrate and insulation layer, and Pt or Au and PEDOT:PSS as conductive films. The fabrication was started with a 5 μm thick substrate layer deposited via CVD (PDS2010 Labcoater from Specialty Coating Systems, Inc., USA) onto a cleaned 4” Si wafer (see Supplementary Fig. S6). Metal traces and the recording sites were realized after spin-coating LOR3b (MicroChemicals, Germany) and AZ nLOF2020 (MicroChemicals, Germany) at 3000 rpm, exposing the resist stack to 40 mJ/cm$^2$ of UV light, and developing in AZ MIF 326 (MicroChemicals, Germany). After surface activation (O${_2}$, 50 W, 80 sccm, 2 min), the metal stack consisting of 10 nm titanium (Ti), 100 nm Pt or Au, and 10 nm Ti was electron beam evaporated (Pfeiffer PLS 570, Pfeiffer Vacuum, Asslar, Germany) with rates of 0.1 nm/s, 0.5 nm/s and 0.1 nm/s, respectively. The wafer were placed in an acetone bath for 3 h to lift off the resists and dipped in AZ MIF 326 to remove residues of LOR3b. Afterwards, a 5 μm insulation PaC layer in combination with Gamma-Methacryloxypropyltrimethoxysilane (A-174 Silane, Specialty Coating Systems, Inc., USA) was deposited. To define the probe shape and to expose the bond pads, the etch mask consisting of 20 μm thick AZ 10XT (MicroChemicals, Germany) was prepared by a double spin-coating scheme (1. layer: 2,400 rpm, 2. layer: 2,100 rpm) and a photolithography process employing exposure to 2,100 mJ/cm$^2$ of UV light and development in AZ 400K (MicroChemicals, Germany). The PaC films were etched with a rate of 0.70 μm/min (CF$_4$:O$_2$, 4:36 sccm, 0.007 mbar, RF 50 W, ICP 500 W, 10 °C, Oxford Plasma Technology RIE reactor, United Kingdom). After etching the Ti etch stop layer (6 nm/min, Ar:O$_2$, 20:20 sccm, 0.035 mbar, RF 50 W, 10 °C), the remaining resist was stripped with AZ 100 Remover (MicroChemicals, Germany) employing ultrasonication. Prior to exposing the recording sites, 2 % (v/v in ultrapure water) Micro90 soap solution (International products cooperation, USA) was spin-coated at 1000 rpm followed by the CVD of a 4 μm thick PaC sacrificial layer^31^. Dry etching of the recording sites was performed as described above by introducing the openings into the insulation and the sacrificial layer. After surface activation, PEDOT:PSS consisting of 93.9 % (v/v) Clevios PH-1000 (Heraeus Holding GmbH, Germany), 5 % (v/v) EG, 1 % (v/v) GOPS, and 0.1 % (v/v) DBSA (Sigma-Aldrich, Germany) was spin-coated three times, once at 3000 rpm and two times at 500 rpm with a 1 min baking step at 90 °C  in between. With the aid of a few drops of water, the PaC sacrificial layer was peeled off and after hard baking at 140 °C  for 1 h, the probes were soaked overnight in water to remove Micro90 residues. At the end, the probes were dry released from the wafer and soldered via flip-chip bonding to custom-made printed circuit boards (PCBs) using the soldering paste NC-31 (AMTECH, USA). The finale PaC based probe soldered to the PCB is shown in Supplementary Fig. S7. The optical validation was carried out with the digital microscope VK-X150 (Keyence Deutschland GmbH, Germany) and a scanning electron microscope (SEM) (Magellan 400, FEI Deutschland GmbH, Germany) at 3 kV acceleration voltage and a current of 50 pA using inlens detection after sputtering iridium at 15 mA for 45 s (Sputter coater K575x, Emitech GmbH, Germany). The detailed fabrication protocol of the second generation is provided in the Supplementary Method S1.

### Insertion system

#### Reducing effective length

Single shanks of the flexible array were classified into long columns considering the slenderness ratio $\lambda$^36,37^:

1 $$\begin{aligned} \lambda =\frac{L_e}{r_x} \quad {\text {with}} \quad L_e=L\cdot K \quad {\text {,}} \quad r_x=\biggl (\frac{I_m}{A}\biggr )^{\frac{1}{2}} \quad {\text {and}} \quad I_m=\frac{1}{12}bh^3 \end{aligned}$$

with $L_e$ the effective length [m], $r_x$ the radius of gyration [m], *L* the column length, $I_m$ the moment of inertia [m$^4$], *A* the cross-sectional area [m$^2$], *b* the column width [m], *h* the column thickness [m], and *K* the effective length constant, which equals 0.7 for a fixed-pinned column. Afterwards, the column’s critical load for buckling $P_{cr}$ was determined using the Euler’s formula^36,37^:

2 $$\begin{aligned} P_{cr}=\frac{\pi ^2 E I_m}{L_e^2} \end{aligned}$$

with *E* the Young’s modulus [Pa].

#### PEG coating

The neural probes were covered with PEG using a custom-made polydimethylsiloxane (PDMS, Sylgard 184, Dow Corning, USA) mold. The mold, the base layer, and the cover sheet were cut from a 120 μm PDMS slap. As shown in the Supplementary Fig. S8, the shanks of the array were aligned between the base and mold by covering the first 200–300 μm from the tip and the assembly was heated up to 80 °C on a hotplate. A granulate of PEG with a molecular weight of 35,000 g/mol (Sigma-Aldrich, Germany) was molten on top of the mold and carefully spread over the four shanks. If needed, the cover sheet was placed on top of the mold to remove excess PEG. After solidifying the PEG at room temperature and peeling off the cover sheet as well as the mold, the array with the PEG coating was released.

#### Insertion experiment into brain phantoms

The brain phantom, an agarose gel, was prepared by mixing 0.06 g agarose powder (A9539-256, Sigma Aldrich, Germany) with 10 ml 0.1 M PBS. The mixture was microwaved a few times at medium level and swirled slowly in between to avoid over-boiling until it became transparent. Then, the solution was cooled down to form the gel. The flexible probes soldered to the PCB and mechanically reinforced by the PEG coating were clamped to a micromanipulator system (Luigs & Neumann, Germany).

### Impedance characterization

The electrochemical impedance of the PEDOT:PSS films was determined with the potentiostat VSP-300 (Bio-Logic Science Instruments SAS, France) using the three electrode configuration featuring the microelectrodes of the neural probes as working electrode, a coiled Pt wire as counter electrode, and a Ag/AgCl electrode as reference electrode. The measurements were performed in 0.1 M PBS by applying a 10 mV sine wave. The mean impedance was calculated by averaging 12 probes from two different fabrication runs. Electrodes with an impedance higher than 11 M$\Omega \cdot$μm$^2$ at 1 kHz ($\approx$35 %) were excluded due to fabrication defects.

### Animal experiments

Electrophysiological recordings were performed on wild-type mice of either sex between 3–12 months of age. Mice were housed under standard conditions in ventilated racks. Experimental protocols were approved by the Landesamt für Natur, Umwelt und Verbraucherschutz NRW (LANUV NRW) (State Office for Nature, Environment and Consumer Protection North Rhine-Westphalia), Postfach 101052, 45610 Recklinghausen, Germany and comply with European Union legislation and recommendations by the Federation of European Laboratory Animal Science. This study was carried out in compliance with the ARRIVE guidelines (https://arriveguidelines.org). In preparation for the recordings, a craniotomy was made above the barrel cortex (n = 3 mice) or the olfactory bulb (n = 5 mice). All described electrophysiological experiments were acute and done under anesthesia. For each animal, 1–3 penetrations were made with the flexible probes as well as the stiff devices depending on the recording quality. The detailed protocol of the animal surgeries is provided in the Supplementary Method S2.

#### Stimulation

*Tactile stimulation* Stimulation was delivered using air puffs directed at the whiskers on the contralateral side of the recorded barrel cortex. Air puffs were delivered in sets of three puffs (duration of 100 ms each, one puff per second), followed by an inter-stimulus interval of 10 s. This cycle was repeated 50 times.

*Odorant stimulation* Odor delivery was achieved using a computer-controlled olfactometer. The following list of odorants was used: ethyl butyrate, methyl valerate, propyl butyrate, isoamyl acetate, 2-Pentanone, ethyl tiglate, sec-butyl acetate, and 2-Hexanone. All odorants were obtained with a 95–99 % purity from Sigma-Aldrich, Germany and were presented as dilutions from saturated vapor in cleaned and humidified air. Odorant concentration was set between 1–2 % from saturated vapor. Odorant stimulation lasted 10 s and was followed by an inter-stimulus interval of 40 s. This cycle was repeated six times for each odorant.

#### In vivo electrophysiology

Recordings were performed using either flexible or stiff (ATLAS Neuroengineering bvba, Belgium or NeuroNexus Technologies, Inc., USA) neural probes. The probes soldered to PCBs were directly connected to a 16 channel headstage (RA16AC or SH16, Tucker Davis Technologie (TDT), USA) that was secured to a micromanipulator (2825B, Thorlabs Inc., USA) with a motorized z-axis. After being amplified and digitized on a preamplifier (RA16PA 16 Channel Medusa Preamp, Tucker Davis Technologie (TDT), USA), detected signals were sent to a digital signal processor (RZ5 Bioamp Processor, TDT, USA). The sampling rate was 24 kHz.

#### Histology

Some of the flexible probes were labeled with DiI (D282, Invitrogen, USA) for postmortem reconstruction of the penetration tracks. The details are provided in the Supplementary Method S3.

#### Data analysis and statistics

After probe insertion, several trials were recorded and used for the subsequent analysis. Amplitude thresholding was utilized to isolate individual spike waveforms. For single spikes excessing the threshold of 3.9 times standard deviations above and below the mean of the filtered trace [5th order Butterworth band-pass (300 Hz–10 kHz)], a 1.5 ms snippet centred on the absolute minimum of the waveform was extracted. Spikes were sorted in MATLAB 2020 (Mathworks Inc., USA) using the package UltraMegaSort2000^49^ and exported to Python 3^50^ for further analysis. SNR was defined as following:

3 $$\begin{aligned} SNR = \frac{A_{mean}}{2 \cdot noise_{RMS}} \end{aligned}$$

with $A_{mean}$ the peak-to-peak spike amplitude of the average waveform and $noise_{RMS}$ the RMS background noise of the associated channel, calculated using 1 s of the filtered trace excluding the peaks. The isolated units were classified as SUA when the clusters consisted of more than 100 individual spikes and when the refractory period violation was less than 1 % for a refractory period of 2.5 ms. The continuous variables signal amplitude, noise and SNR were compared employing the Mann-Whitney-U Test. Categorical variables such as yield of SUA were compared utilizing the chi-square test. If p-values were lower than 0.05 (*p* $\le$ 0.05), differences in studied variables were considered to be statistically significant. All *p*-values less than 0.0001 (*p* $\le$ 0.0001) were summarized with four asterisks (****).

## Supplementary Information

Supplementary Information.

## Data Availability

The datasets generated and analysed during the current study are available from the corresponding author on reasonable request.
