# Supplementary material for: Minimally-invasive insertion strategy and in vivo evaluation of multi-shank flexible intracortical probes
Source: Sci Rep. 2021 Sep 23;11:18920. doi: 10.1038/s41598-021-97940-x (PMC8460634; doi:10.1038/s41598-021-97940-x)
Supplement: Supplementary file 1 — Supplementary Information. [file 41598_2021_97940_MOESM1_ESM.pdf]

Supplementary Information

Minimally-invasive insertion strategy and *in vivo* evaluation of multi-shank flexible intracortical probes

Kagithiri Srikantharajah[1,5], Renata Medinaceli Quintela[2,+], Kerstin Doerenkamp[3,+], Björn M. Kampa[3,4], Simon Musall[1], Markus Rothermel[2], and Andreas Offenhäusser[1,5,*]

[1]Bioelectronics, Institute of Biological Information Processing-3, Forschungszentrum Jülich, Jülich, Germany
[2]Institute for Physiology and Cell Biology, University of Veterinary Medicine, Foundation, Hannover, Germany
[3]Department of Neurophysiology, Institute for Biology II, RWTH Aachen University, Aachen, Germany
[4]JARA BRAIN, Institute for Neuroscience and Medicine, Forschungszentrum Jülich, Jülich, Germany
[5]RWTH Aachen University, Aachen, Germany
[*]a.offenhaeusser@fz-juelich.de
[+]these authors contributed equally to this work

Supplementary Figures

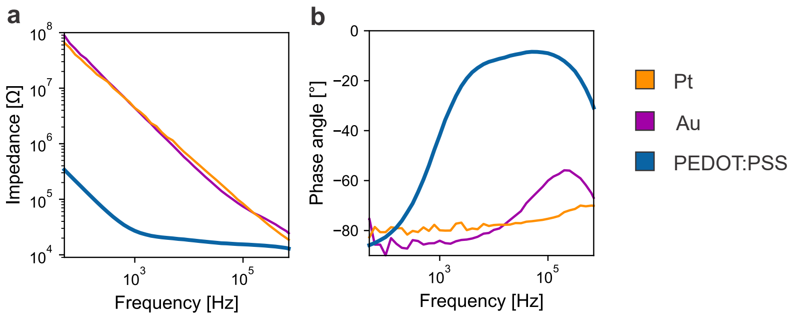

Figure S1. Exemplary impedance spectra of PEDOT:PSS in comparison to bare Pt and Au microelectrodes with a GSA of 113 μm^2.

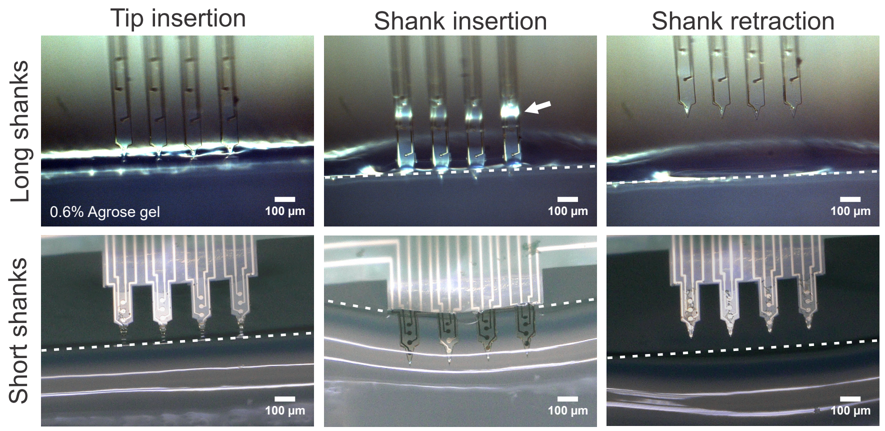

Figure S2. Insertion mechanism of flexible penetrating probes. 2 mm long shanks failed to penetrate the brain phantom due to buckling (arrow). On the contrary, short shanks with a length of 300 μm successfully penetrated the brain phantom. Dashed line indicates surface of the 0.6 % agarose gel brain phantom.

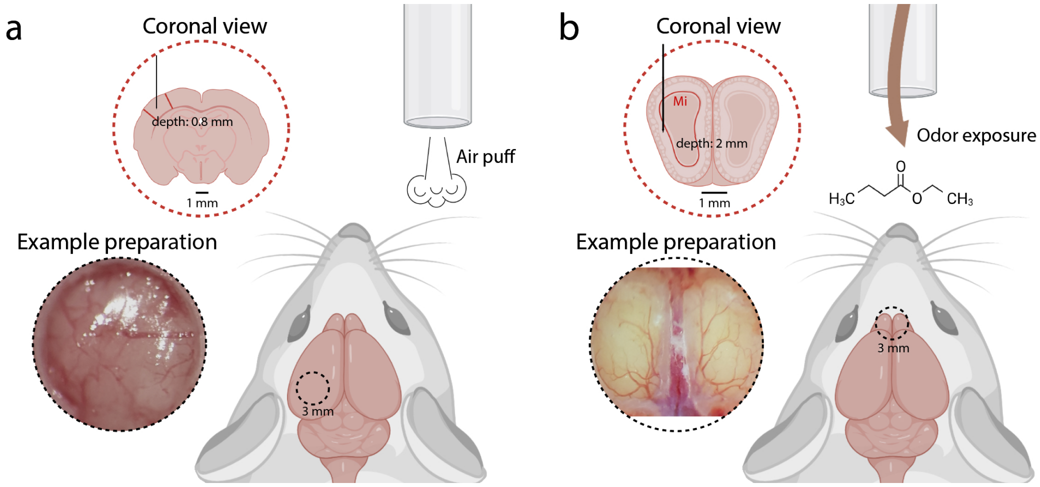

Figure S3. Overview of the two target brain regions: a) barrel cortex and b) olfactory bulb with the different dorsoventral depths (see coronal view). Mi: mitral cell layer. Image created with BioRender.com.

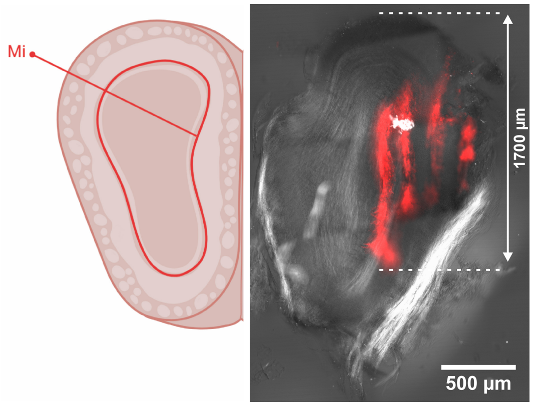

Figure S4. Penetration tracks stained with DiI (red) depicting an exemplary insertion of the flexible intracortical probes into the mouse olfactory bulb. The four single shanks of the array could be distinguished. Mi: mitral cell layer. Drawing created with BioRender.com.

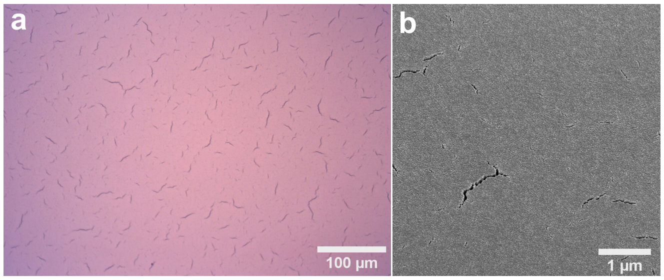

Figure S5. Electron beam evaporation of 100 nm Pt on PaC films resulted in a micro-cracked morphology.

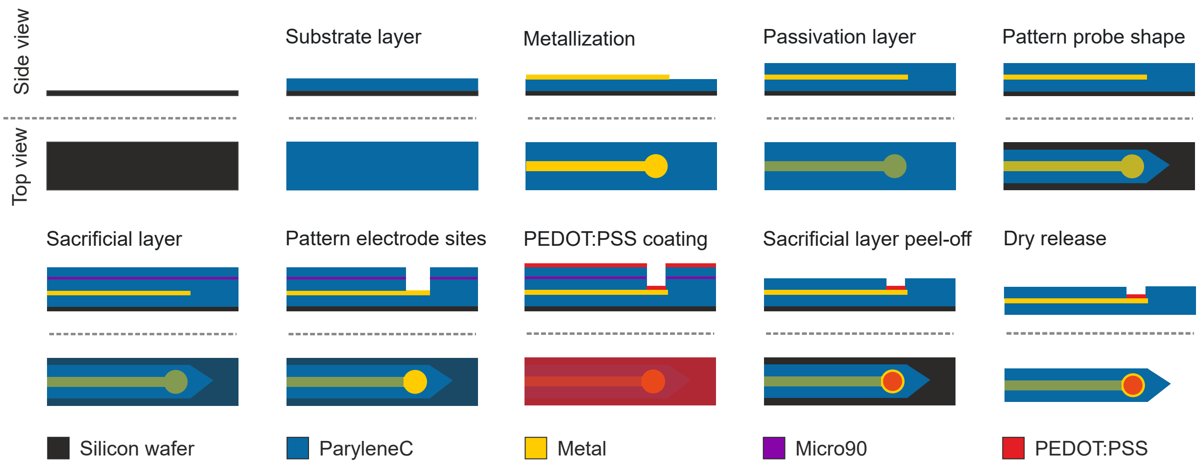

Figure S6. Microfabrication flow for ParyleneC (PaC) based devices with PEDOT:PSS coating. Starting from a plain Silicon wafer, the first layer of PaC was deposited via chemical vapour deposition (CVD) and used as substrate layer. Physical vapour deposition (PVD) was utilized to evaporate the metal stack. After depositing the passivation layer, the probe shape was patterned via reactive ion etching (RIE). A release layer consisting of Micro90 soap solution and a PaC sacrificial layer were applied before exposing the electrode sites. After spin-coating and soft baking the PEDOT:PSS solution, the sacrificial layer was peeled off and the final probes were dry released.

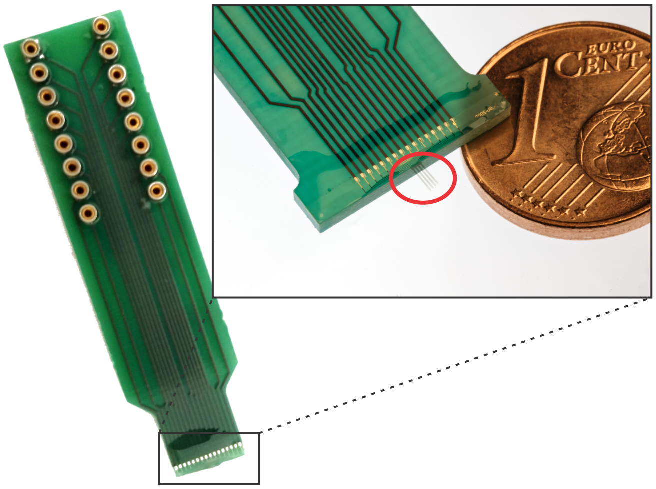

Figure S7. The final device comprising a custom-made printed circuit board with 16 channels and a PaC based probe (red circle).

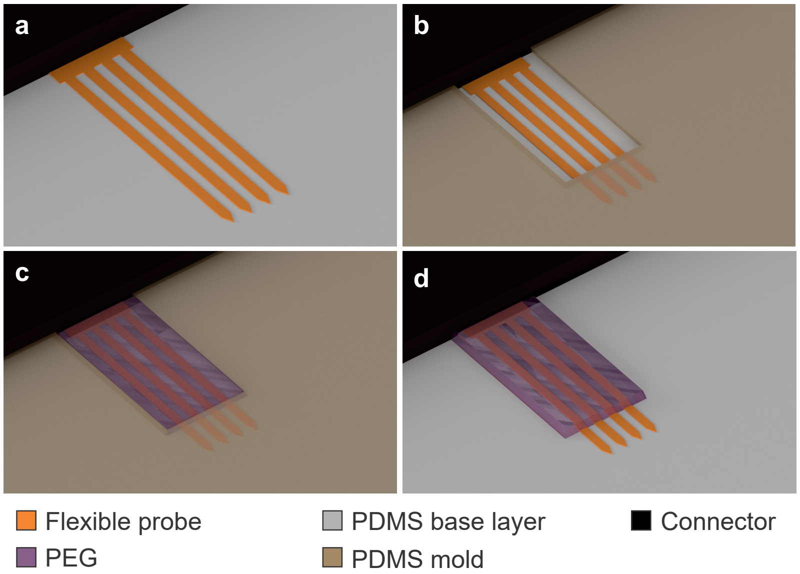

Figure S8. Coating approach to temporary reduce the effective shank length. Flexible probe was aligned between PDMS base layer (a) and 120 µm thick PDMS mold (b) covering the first 200-300 µm from the shank tips. c) Casting of polyethylene glycol (PEG) (35,000 g/mol). d) Removal of PDMS mold after solidifying the PEG.

Supplementary Tables

Insertion system	Flexible probe	Probe cross-section [μm^2]	Implantation footprint [μm^2]
**PEG [This work]**	**A-MEA-16**	**1,000**	**1,000**
PEG[1]	PaC/SU-8	1,800	9,000
Si[2]	PI	5,360	16,000
PVA/PLGA[3]	PaC	1,520	33,440
Maltose[4]	PI	2,000	62,000
PEG[5]	PaC	4,800	87,400

Table S1. Comparison of implantation footprints from different insertion systems. Probe cross-section is obtained by multiplying shank width and thickness. For the implantation footprint, dimensions of neural implant and insertion aid were summed.

	Flexible array [This work]	NeuroNexus	ATLAS
Insulation material	PaC	Si	Si
Number of shanks	4	1	1
Shank thickness [μm]	10	15	50
Shank width [μm]	100	125-200	75
Shank length [mm]	2	5	6
Inter-shank distance [μm]	100		
Electrodes per shank [μm]	4	16	16
Electrode pitch [μm]	200	50	100
Electrode area [μm^2]	113	413	491
Electrode coating	PEDOT:PSS	IrOx	IrOx
Electrode impedance [MΩ·μm^2]	2.93±0.73 (n=125)		112.93±4.91 (n=16)
Tip angle [°]	30-40		pointy tip feature

Table S2. The critical dimensions of the neural probes used within this work. The probe A1x16-5mm-50-413 from NeuroNexus Technologies, Inc., USA and the probe E16+R-100-S1-L6 NT from ATLAS Neuroengineering bvba, Belgium were used. The impedances at 1 kHz are given as mean±std.

Probe type	Probe cross-section [μm^2]	Number of electrodes	Cross-section per electrode [μm^2]
**PaC - Second generation [This work]**	**945**	**8**	**118**
**PaC - First generation [This work]**	**1,000**	**4**	**250**
PaC[3]	1,520	6	253
Si - A1x16-5mm-50-413[a]	1,875	16	117
PaC[6]	2,600	8	325
PaC[7]	3,600	8	450
Si - E16+R-100-S1-L6 NT[b]	3,750	16	234
PaC[8]	3,900	64	61

Table S3. Comparison of flexible probes' dimensions. The cross-section is obtained by multiplying shank width and thickness. For cross-section per electrode, number of electrodes per shank were considered. a) NeuroNexus Technologies, Inc., USA, b) ATLAS Neuroengineering bvba, Belgium.

Supplementary Methods

Method S1: Microfabrication

The double-metal-layer fabrication of the second generation of flexible devices was started with the chemical vapour deposition of a 5 µm thick PaC film as substrate layer on a cleaned 4" Si wafer. In the first metallization step, the metal traces with a minimum line width and pitch of 5 µm were evaporated via electron beam evaporation and structured using a lift off process. Due to the small dimensions of the interconnects, the photolithography process was changed from the double resist system (used for the first generation) to using only AZ nLOF2020. Furthermore, the exposure dose was adjusted to 17 mJ/cm^2. A 500 nm thick PaC was deposited as insulation between the two metal layers. Openings with a diameter of 5 µm were introduced in the interlayer over the feedline ends via reactive ion etching. Recording sites and bond pads were defined in the second metallization step as described above. To ensure electrical connectivity between the two conductive layers, the thickness of the second metal stack was matched to the thickness of the PaC interlayer. Thus, the metal stack consisting of 10 nm titanium and 500 nm gold were evaporated. After the deposition of a 5 µm thick PaC film as passivation layer, the probe shapes were structured and the recording sites were exposed within the final etching step. Finally, the probes were dry released from the carrier wafer and soldered via flip-chip bonding to custom-made printed circuit boards.

Method S2: Animal surgeries

Barrel cortex: In preparation for the recordings, mice underwent two surgeries. One week prior to the experiments, a headholder was implanted onto the skull of the mice. For this, mice were anesthetized using inhaled isoflurane (5 % for induction, 1.5 – 2.5 % during surgery). Mice were placed on an electrical heating blanket (TC200 Temperature controller, Thorlabs GmbH, Germany) for the duration of the surgery. For analgesia, mice were injected with buprenorphin (0.1 mg/kg, subcutaneous, Buprenovet, Bayer AG, Germany). Additionally, bupivacain (PUREN Pharma GmbH & Co. KG, Germany) was used as local anesthetic and was injected at the incision site. The hair above the skull was removed and the skin sterilized using iodine. A small incision was made along the midline. The skull underneath was cleaned, and the muscles gently pushed aside and fixed in place using vetbond. A small craniotomy was made above the cerebellum and a ground pin was implanted. A custom designed headbar was placed above the skull and both the headbar and ground pin were fixed in place using dental cement. Any remaining exposed skull area was covered in vetbond for protection. 9 mg/l buprenorphin and 1 ml/l baytril (Bayer AG, Germany) were added to the drinking water after surgery until the experiment. On the day prior to the experiment, the mice underwent a second surgery where a craniotomy was made above the barrel cortex (AP:-1, ML:-3, diameter:3 mm). Again, isoflurane was used for anesthesia and buprenorphin was injected for analgesia. Additionally, prednisolonacetat was injected (1.2 mg, intramuscular, CP-Pharma, Germany) to prevent brain swelling. For the recording, mice were lightly anesthetized with inhaled isoflurane (0.5 – 1 %) and headfixed. Mice were placed on an electrical heating pad for the duration of the experiment. Prednisolonacetat was injected to prevent brain swelling. Slits were introduced to the dura that served as entry points for the probe shanks.

Olfactory bulb: Extracellular recordings of olfactory bulb output activity were performed as previously described[9]. Briefly, mice were anesthetized by intraperitoneal injection of pentobarbital (50 mg/kg, Narcoren (16g/100 ml), Boehringer Ingelheim Vetmedica GmbH, Germany). Further doses of pentobarbital were administered via a shunt placed intraperitoneally into the mouse's abdominal region. Mice were placed on a heating blanket with circulating water (T/pump Professional, Stryker, USA) to keep core body temperature at about 37 °C for the duration of the experiment. Bupivacain (PUREN Pharma GmbH & Co. KG, Germany) was used as local anesthetic and was injected at all incision sites. Mice underwent a double tracheotomy and an artificial sniff paradigm was used to reliably control air and odorant inhalation independent of respiration[10]. Following the double tracheotomy, mice were fixed in a custom-built stereotaxic frame. A dental drill was used to first thin and later remove the bone covering both olfactory bulbs. Finally, with the help of fine tweezers, the dura was also removed to permit probe penetration.

Method S3: Histology

The DiI solution was prepared by diluting the powder (D282, Invitrogen, USA) in ethanol (50 mg/ml)[11]. The flexible probes were labelled with DiI by dipping several times into the dye solution and allowing them to dry in air for a few seconds between dips. After successful insertion, the probes coated with DiI were left in the brain for roughly one hour for postmortem reconstruction of the penetration tracks. At the end of the acute recordings, the mice were overdosed with pentobarbital (Narcoren (16g/100 ml), Boehringer Ingelheim Vetmedica GmbH, Germany) and perfused with PBS and 4 % (v/v) paraformaldehyde (Carl Roth GmbH & Co. KG, Germany) in PBS. Perfused mouse heads were stored in paraformaldehyde in PBS at least for 24 hours before extracting the brain. After extraction, the brains were either stored in paraformaldehyde solution or in 30 % (w/v) sucrose solution. Sectioning was performed by embedding the brain in 3 % low-melting point agarose, and 100-200 µm thick coronal slices were prepared using a vibratome (752/M vibroslice; Campden Instruments Limited, UK). Dil fluorescence was evaluated using a confocal-laser scanning microscope (TCS SP2; Leica GmbH, Germany). Confocal evaluations of fluorescence were

made maximal three months after the experiment was performed.

References

1. Seo, K. J. *et al.* Transparent, flexible, penetrating microelectrode arrays with capabilities of single-unit electrophysiology. *Adv. Biosyst.* **1800276**, DOI: 10.1002/adbi.201800276 (2019).
2. Felix, S. *et al.* Removable silicon insertion stiffeners for neural probes using polyethylene glycol as a biodissolvable adhesive. *Proc. Annu. Int. Conf. IEEE Eng. Medicine Biol. Soc.* 871–874, DOI: 10.1109/EMBC.2012.6346070 (2012).
3. Pas, J. *et al.* A bilayered PVA/PLGA-bioresorbable shuttle to improve the implantation of flexible neural probes. *J. Neural Eng.* **15**, 1–10, DOI: 10.1088/1741-2552/aadc1d (2018).
4. Xiang, Z. *et al.* Ultra-thin flexible polyimide neural probe embedded in a dissolvable maltose-coated microneedle. *J. Micromechanics Microengineering* **24**, 1–11, DOI: 10.1088/0960-1317/24/6/065015 (2014).
5. Lecomte, A. *et al.* Silk and PEG as means to stiffen a parylene probe for insertion in the brain: Toward a double time-scale tool for local drug delivery. *J. Micromechanics Microengineering* **25**, 1–12, DOI: 10.1088/0960-1317/25/12/125003 (2015).
6. Wang, X. *et al.* A parylene neural probe array for multi-region deep brain recordings. *J. Microelectromechanical Syst.* **29**, 499–513, DOI: 10.1109/JMEMS.2020.3000235 (2020).
7. Hara, S. A. *et al.* Long-term stability of intracortical recordings using perforated and arrayed Parylene sheath electrodes. *J. Neural Eng.* **13**, 1–17, DOI: 10.1088/1741-2560/13/6/066020 (2016).
8. Scholten, K., Larson, C. E., Xu, H., Song, D. & Meng, E. A 512-Channel Multi-Layer Polymer-Based Neural Probe Array. *J. Microelectromechanical Syst.* **29**, 1054–1058, DOI: 10.1109/JMEMS.2020.2999550 (2020).
9. Medinaceli Quintela, R. *et al.* Dynamic impairment of olfactory behavior and signaling mediated by an olfactory corticofugal system. *J. Neurosci.* 863–876, DOI: 10.1523/JNEUROSCI.2667-19.2020 (2020).
10. Wachowiak, M. & Cohen, L. B. Representation of odorants by receptor neuron input to the mouse olfactory bulb. *Neuron* **32**, 723–735, DOI: 10.1016/S0896-6273(01)00506-2 (2001).
11. DiCarlo, J. J., Lane, J. W., Hsiao, S. S. & Johnson, K. O. Marking microelectrode penetrations with fluorescent dyes. *J. Neurosci. Methods* **64**, 75–81, DOI: 10.1016/0165-0270(95)00113-1 (1996).
